# An extensive image dataset for deep learning-based classification of rice kernel varieties in Bangladesh

**DOI:** 10.1016/j.dib.2024.111109

**Published:** 2024-11-06

**Authors:** Md Tahsin, Md. Mafiul Hasan Matin, Mashrufa Khandaker, Redita Sultana Reemu, Mehrab Islam Arnab, Mohammad Rifat Ahmmad Rashid, Md Mostofa Kamal Rasel, Mohammad Manzurul Islam, Maheen Islam, Md. Sawkat Ali

**Affiliations:** Department of Computer Science and Engineering, East West University, Aftabnagar, Dhaka, Bangladesh

**Keywords:** Image Classification, Rice Variety Classification, Deep Learning Precision, Rice Image

## Abstract

This article introduces a comprehensive dataset developed in collaboration with the Bangladesh Institute of Nuclear Agriculture (BINA) and the Bangladesh Rice Research Institute (BRRI), featuring high-resolution images of 38 local rice varieties. Captured using advanced microscopic cameras, the dataset comprises 19,000 original images, enhanced through data augmentation techniques to include an additional 57,000 images, totaling 76,000 images. These techniques, which include transformations such as scaling, rotation, and lighting adjustments, enrich the dataset by simulating various environmental conditions, providing a broader perspective on each variety. The diverse array of rice strains such as BD33, BD30, BD39, among others, are meticulously detailed through their unique characteristics—color, size, and utility in agriculture—providing a rich resource for research. This augmented dataset not only enhances the understanding of rice diversity but also supports the development of innovative agricultural practices and breeding programs, offering a critical tool for researchers aiming to analyze and leverage rice genetic diversity effectively.

Specifications Table

**Table:** 

Subject	Computer Science
Specific subject area	Machine Learning, Deep Learning, Pattern Recognition, Computer Vision and Digital Image Processing.
Type of data	Image
Data collection	This dataset encompasses 38 unique rice varieties including, BD33, BD30, BD39, BD56, BD93, BD91, BD49, BD51, BD52, BD76, BD95, BD57, BD87, BD70, BD85, BD72, BD79, BD75, Binadhan7, Binadhan8, Binadhan10, Binadhan11, Binadhan12, Binadhan14, Binadhan16, Binadhan17, Binadhan19, Binadhan20, Binadhan21, Binadhan23, Binadhan24, Binadhan25, Binadhan26, BR22, BR23, BRRI67, BRRI74, and BRRI102 and the rice kernels were collected from two different Agricultural Research Centres of Bangladesh. Collectively 500 pictures were captured of each rice variety. It has 19000 original images and 76000 augmented images. Digital microscope camera was used for capturing images. The image capturing time was between 15 January and 28 February 2024.
Data source location	Location: Bangladesh Institute of Nuclear Agriculture (BINA) and Bangladesh Institute of Rice Research Institute (BRRI) Country: Bangladesh Latitude and Longitude: 24.7351° N, 90.4286° E (BINA) 23°59′29.3″N 90°24′25.2″E (BRRI)
Data accessibility	Repository name: Mendeley DataData identification number: 10.17632/2fgv99854n.1Direct URL to data: https://data.mendeley.com/datasets/2fgv99854n/1Instructions for accessing these data: - Click on the ‘Files’ section of the dataset page. - Locate the "Original.zip" for unaltered images and "Augmented.zip" for images that have undergone augmentation. Click on each file name to initiate the download. Original.zip: Contains folders named according to rice variety, each holding 500 high-resolution original images of rice kernels. Augmented.zip: Contains similarly organized folders, each with 2,000 images that have been augmented to include variations in angle, lighting, and orientation to aid in robust machine learning training.
Related research article	None.

## 1 Value of the Data

• The dataset provides detailed images and specific measurements of various rice varieties, capturing essential attributes such as grain size, color, and texture [1]. These detailed parameters are critical for selecting high-quality seeds and optimizing yields. Breeders, for example, can utilize this data to choose seeds with optimal traits, ensuring the consistency and purity of rice varieties and minimizing cross-contamination, thereby improving the overall quality and uniformity of rice production.
• Furthermore, this dataset enriches agricultural practices by offering comprehensive data on the morphological and spectral properties of different rice types. Researchers can leverage these insights to refine cultivation techniques, enhance resistance to pests and diseases, and tailor growing conditions to suit distinct rice varieties, enhancing the effectiveness of crop management [2].
• Additionally, this dataset provides a robust platform for testing and refining deep learning algorithms. With images from 38 closely related rice varieties, it serves as an exacting standard for evaluating image classification models, which is particularly beneficial for those in machine learning and computer vision fields.
• Serving as a benchmark, the dataset also allows for the fair assessment and comparison of image classification methods. Researchers can use this dataset to validate and refine their models, ensuring that their approaches are effective against a well-characterized and diverse array of data.
• This dataset can prove invaluable for various stakeholders, including rice breeders, agricultural researchers, and industry professionals [1]. It equips breeders with the tools to create new rice varieties with enhanced characteristics, aids researchers in devising novel crop management techniques, and provides industry professionals with insights to improve product quality and pioneer new production technologies [3].

## 2 Background

Rice stands as the cornerstone of agriculture and diet in Bangladesh and is a vital staple for half of the global population [1]. Recognizing its central role in food security and economic stability, there is a growing need to precisely identify and classify rice varieties to combat adulteration and enhance breeding programs. Recent advances have led to a proliferation of genetically modified rice strains, compounding the challenge of distinguishing between varieties based on traditional physical characteristics such as shape, color, and texture. This is exacerbated by the limitations of existing image datasets, which often lack the diversity and depth needed to capture the nuanced differences between these varieties [2]. To address these challenges, this study introduces a meticulously curated dataset, captured using high-resolution microscopic imaging, featuring 38 distinct rice varieties from Bangladesh. This dataset is not only a step towards refining classification methods but also serves as a critical tool for advancing agricultural practices and developing innovative crop management techniques, thus bolstering the efforts towards enhanced food security and sustainable agricultural development.

## 3 Data Description

This article provides a new and large rice image dataset that includes 38 different varieties [5,6]: BD33, BD30, BD39, BD56, BD93, BD91, BD49, BD51, BD52, BD76, BD95, BD57, BD87, BD70, BD85, BD72, BD79, BD75, Binadhan7, Binadhan8, Binadhan10, Binadhan11, Binadhan12, Binadhan14, Binadhan16, Binadhan17, Binadhan19, Binadhan20, Binadhan21, Binadhan23, Binadhan24, Binadhan25, Binadhan26, BR22, BR23, BRRI67, BRRI74, and BRRI102. We have used the name suggested by Bangladesh Rice Research Institute. Under the supervision of a specialist, we used two microscope cameras to take images. The dataset has 19000 original images and 76000 augmented images. Each type of rice has 500 RGB microscopic photos, which are kept in separate files named after the rice species. Every image is in JPG format and has 640 × 480 pixels as its dimensions.

**Fig. 1:**
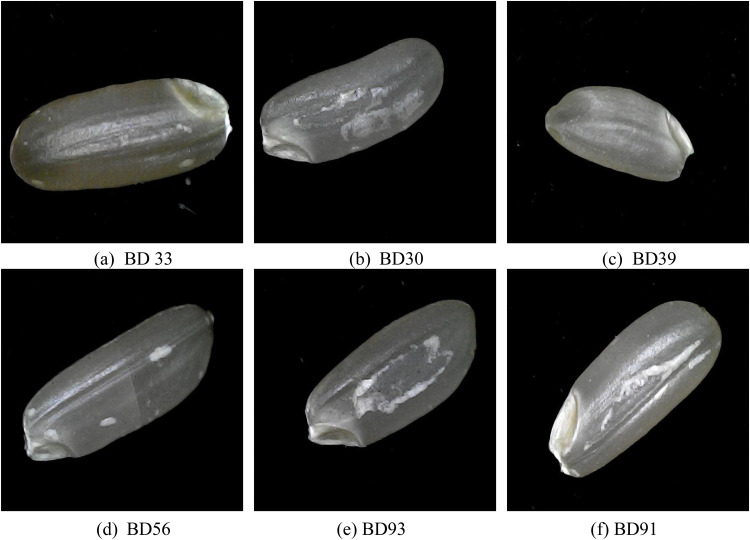
Images of selected rice varieties highlighting key physical traits. Top row: (a) BD33 - features short, thick grains with a robust plant structure, (b) BD30 - medium-thin, white grains with upward-pointing leaves, (c) BD39 - long, thin grains supported by a strong stem. Bottom row: (d) BD56 - has dark green, erect leaves with red-hued grains, (e) BD93 - a Ufshi variety producing medium-coarse red grains, (f) BD91 - a tall variety with submergence tolerance and medium-thick grains.

The BD rice varieties, including BD33, BD30, BD39, and others, each showcase unique characteristics suited for different growing conditions and purposes (Fig. 1). BD33 [5], for example, grows to a height of 100 cm with strong, thick stems and short, thick grains, and has wider leaves with light brown folds near the tip. It has a lifespan of 118 days. BD30 [5] reaches 120 cm, featuring medium-thin, white grains and light-sensitive leaves that gradually point upward; it matures in 145 days. BD39 [5], slightly shorter at 106 cm, produces long, thin grains with wider and steeper leaves, maturing in 120 days. BD56 and BD93 are both resilient types, with BD56 showing moderate flood tolerance, growing to 115 cm, and producing long, thick, white rice with a lifespan of 135 days in dry conditions, extending to 160 days in floods. BD93 [5], a high-yielding Ufshi variety, grows up to 117 cm and produces red, medium-coarse grains with 26.1% amylose and 7.5% protein, maturing in 134 days [5].

The taller BD91 [5] variety reaches up to 190 cm and features strong, upright stems with dark green leaves, making it highly tolerant of waterlogged conditions. Its grains are medium-thick and light brown, with a lifespan of 152–156 days. Shorter varieties like BD49 and BD51 are better suited to flash-flood-prone areas, with BD49 at 100 cm producing medium-coarse white grains containing 8.5% protein, while BD51, at 90 cm, yields transparent white grains with low light sensitivity and a lifespan of 140–145 days. Flood-tolerant varieties BD52 and BD76 also stand out; BD52 reaches 116 cm, producing medium-coarse white rice with a lifespan of 140–145 days, extending to 155–160 days during flash floods, while BD76, growing to 140 cm, produces grains weighing 25.6 grams per 1000 kernels and matures in 153 days. Nutritional varieties like BD95 and BD57 offer high protein and amylose content, with BD95 reaching 120 cm and producing red, medium-coarse grains, while BD57, at 110–115 cm, yields thin, straw-colored grains with a brief lifespan of 100–105 days. Specialty types, such as fragrant BD70 (125 cm tall), and zinc-rich BD72 (116 cm tall), are also notable for their unique grain characteristics, offering long, thin grains and a lifespan of 125–130 days. BD79 and BD75 share Ufshi traits, with heights of 112 cm and 101–110 cm respectively, supported by strong stems that provide resilience and nutritional value over a 110–115-day lifespan [5].

**Fig. 2:**
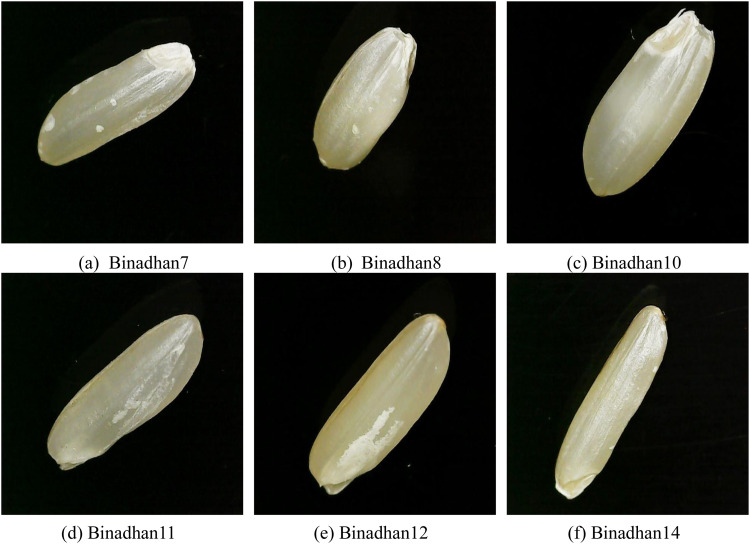
Images of selected Binadhan rice varieties, each adapted for specific environmental conditions and growth characteristics. Top row: (a) Binadhan7 - an early-ripening Aman variety with strong, thick stems and high amylose content, (b) Binadhan8 - salt-tolerant Boro variety suitable for saline environments, (c) Binadhan10 - another salt-tolerant Boro type. Bottom row: (d) Binadhan11 - flood-tolerant Aman variety with resilience for submersion, (e) Binadhan12 - a high-yielding flood-resistant variety, (f) Binadhan14 - Boro type with high temperature tolerance.

The Binadhan rice varieties—including Binadhan7, Binadhan8, Binadhan10, and others—each possess specific traits that make them suitable for distinct environmental conditions (Fig. 2). Binadhan7, an early-ripening Aman variety with a lifespan of 110–120 days, has thick, strong stems and broad, dark green leaves that provide resistance to lodging, producing long, bright-colored grains with a high amylose content of 24–25%, which adds to its marketability. Binadhan8 and Binadhan10 are salt-tolerant Boro varieties that can endure salinity levels of 8–14 ds/m depending on their growth stage, maturing within 130–135 days [5]. Flood-tolerant varieties like Binadhan11 and Binadhan12 are well-suited for areas prone to flash floods, with lifespans of 110–115 and 125–130 days, respectively, and the ability to survive submersion for up to 25 days. Binadhan14, another Boro type, is recognized for its high-temperature tolerance, maturing in about 120–130 days. Binadhan16, with a short lifespan of 100–105 days, produces high yields of long, thin grains, making it ideal for Aman season farming [5].

For regions facing drought, Binadhan17 provides resilience with 30% less water requirement and a brief growing period of 112–118 days. Binadhan19 is suited for rain-dependent Aush and Aman seasons, particularly in areas like Barendra and hilly regions, with a lifespan of 95–105 days. Its growth can resume after drought conditions, making it reliable for water-scarce environments. Nutrient-rich varieties like Binadhan20, which contains high levels of zinc and iron, have long, red grains and mature within 125–130 days. Binadhan21, an Aush variety, yields long, thin, white grains that cook to a fluffy texture, with a lifespan of 100–105 days. Binadhan23 is adapted for tidal, saline, and flood-prone areas, tolerating salinity up to 8 ds/m and 15-day submersion, and maturing in 115–125 days. High-yielding Boro varieties like Binadhan24 and Binadhan25, with lifespans of 143–148 and 138–148 days respectively, offer strong, upright structures and neat, export-quality grains, while Binadhan26, an Aman variety resistant to bacterial leaf blight (BLB) due to Xa4 and xa5 genes, matures in 115–120 days and provides both high amylose and protein content, enhancing its durability and yield potential [5].

**Fig. 3:**
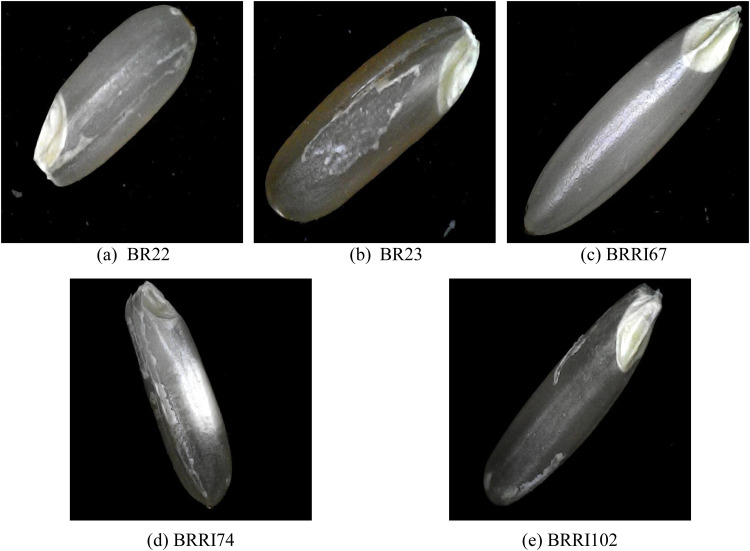
Images of selected BR rice varieties, each showcasing unique adaptations for varied growing conditions. Top row: (a) BR22 - characterized by thick, translucent grains and resistance to tungro and sheath blight, (b) BR23 - long, slender grains with tolerance to salinity and blast, (c) BRRI67 - a high-yielding variety with salinity tolerance. Bottom row: (d) BRRI74 - high-yielding, sturdy plant with medium-thick white grains rich in zinc, (e) BRRI102 - features long, thin, straw-colored grains, with high zinc and amylose content.

The BR rice varieties—BR22, BR23, BRRI67, BRRI74, and BRRI102— as illustrated in Fig. 3, are designed for specific resistance traits, growth characteristics, and nutritional values, making them adaptable for diverse agricultural needs [5]. BR22, with a height of 125 cm, produces short, bold, and translucent grains. It is resistant to tungro and sheath blight and shows moderate tolerance to the yellow stem borer. BR23 is slightly shorter at 105 cm, yielding clean, long, and slender white grains. This variety has moderate tolerance to sheath blight and white-backed plant hopper, along with resilience against blast and partial tolerance to salinity and water stagnation [5].

BRRI67, with a height of 100 cm, is specifically bred for high salinity tolerance. It produces medium-thin, white, neat rice grains and has a lifespan of 140–150 days, making it a high-yielding variety in saline-affected areas [6]. BRRI74, another high-yielding type, is slightly shorter at 92 cm, with a sturdy structure that prevents it from falling. This variety produces medium-thick white grains with a scratchy tip, containing 8.3% protein and 24.2 mg/kg of zinc, with a growth period of 145–147 days [6].

Lastly, BRRI102 integrates many modern Ufshi rice traits and resembles BRRI rice29 in its vegetative phase, with steep, broad, and green leaves. Growing up to 103 cm, BRRI102 yields long, thin, straw-colored grains with 25.5 mg/kg of zinc, 28% amylose, and 7.5% protein. With a lifespan of 150 days, it offers both nutritional value and durability, enhancing its suitability for diverse growing conditions. Each of these varieties provides specific advantages in terms of resistance, yield, and grain quality, supporting productive and resilient rice cultivation [6].

The rice varieties dataset is freely accessible in the Mendeley repository [4]. The dataset consists of two distinct zip files: “Original.zip'' and “Augmented.zip”. Each file contains 38 sub-folders for organizing the data. Every class included in the dataset has incomparable and exceptional features in order to make the images distinguishable by machine learning and deep learning models. Table 2 present the folder structure of the dataset.

**Table 2:** Folder Structure of the dataset.

Sl.	Name	Folder name	Original images	Augmented images
1	**BD33**	1_BD33	500	2000
2	**BD30**	2_BD30	500	2000
3	**BD39**	3_BD39	500	2000
4	**BD56**	4_BD56	500	2000
5	**BD93**	5_BD93	500	2000
6	**BD91**	6_BD91	500	2000
7	**BD49**	7_BD49	500	2000
8	**BD51**	8_BD51	500	2000
9	**BD52**	9_BD52	500	2000
10	**BD76**	10_BD76	500	2000
11	**BD95**	11_BD95	500	2000
12	**BD57**	12_BD57	500	2000
13	**BD87**	13_BD87	500	2000
14	**BD70**	14_BD70	500	2000
15	**BD85**	15_BD85	500	2000
16	**BD72**	16_BD72	500	2000
17	**BD79**	17_BD79	500	2000
18	**BD75**	18_BD75	500	2000
19	**Binadhan7**	19_Binadhan7	500	2000
20	**Binadhan8**	20_Binadhan8	500	2000
21	**Binadhan10**	21_Binadhan10	500	2000
22	**Binadhan11**	22_Binadhan11	500	2000
23	**Binadhan12**	23_Binadhan12	500	2000
24	**Binadhan14**	24_Binadhan14	500	2000
25	**Binadhan16**	25_Binadhan16	500	2000
26	**Binadhan17**	26_Binadhan17	500	2000
27	**Binadhan19**	27_Binadhan19	500	2000
28	**Binadhan20**	28_Binadhan20	500	2000
29	**Binadhan21**	29_Binadhan21	500	2000
30	**Binadhan23**	30_Binadhan23	500	2000
31	**Binadhan24**	31_Binadhan24	500	2000
32	**Binadhan25**	32_Binadhan25	500	2000
33	**Binadhan26**	33_Binadhan26	500	2000
34	**BR22**	34_BR22	500	2000
35	**BR23**	35_BR23	500	2000
36	**BRRI67**	36_BRRI67	500	2000
37	**BRRI74**	37_BRRI74	500	2000
38	**BRRI102**	38_BRRI102	500	2000

**Total =**			**19000**	**76000**

## 4 Experimental Design, Materials and Methods

**Fig. 4:**
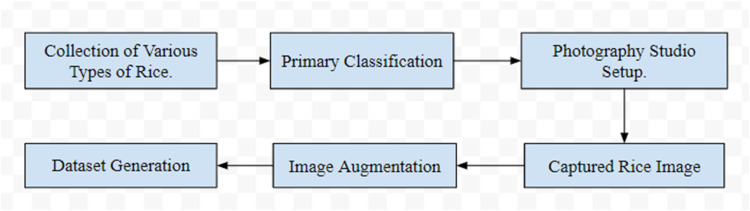
Workflow for the rice varieties image dataset generation.

The process of acquiring images for each type of rice variety has been managed through a hypothetical workflow depicted in Fig. 4. Researchers in Bangladesh have followed a well-organized and methodical process, emphasizing on the acquisition of high-quality, multidimensional, and extensive information. After obtaining images of each rice crop, they have been labeled by agricultural experts alongside agronomists in a meticulously planned manner.

The dataset has been pre-processed to ensure it is in the optimal format for training machine learning models, with images adjusted to various proportions and shapes while extraneous background elements were eliminated. Collaborative efforts with institutions skilled in aggregating crucial, advanced information about rice varieties in Bangladesh have led to the collection of ideal samples. These institutions have curated and maintained a selection of specific rice varieties under optimal conditions, enriching the dataset's diversity with samples showcasing a range of colors, shapes, and sizes. This extensive collection is perfectly suited for advanced research. In January 2024, high-resolution images were captured using dual microscope cameras under 38 distinct harvesting conditions, each selected to highlight the unique characteristics of the rice types. These images were meticulously taken in a dust-controlled environment to ensure clarity and precision, utilizing optimal lighting and camera angles to accentuate key features of each variety. The research institutions have meticulously labeled and categorized each sample to maintain high data quality, supporting the development of robust machine learning models.

A black cloth background was utilized to ensure precise focus on each crop. Images were saved in JPG format and subsequently organized into folders categorized by rice species. Before finalizing the folders, any extraneous or irrelevant images were removed. Each of the 38 subfolders contains 500 RGB microscopic images, clear and accurately captured, summing up to 19,000 images in total. All images and folders were meticulously labeled. Data augmentation techniques were then applied to meet the extensive image requirements of machine vision-based deep learning models. Augmentation processes included scaling, shifting, shearing, cropping, flipping, and brightness adjustments, utilizing techniques like bilinear and nearest-neighbor interpolation for scaling and histogram equalization for brightness adjustments.

**Fig. 5:**
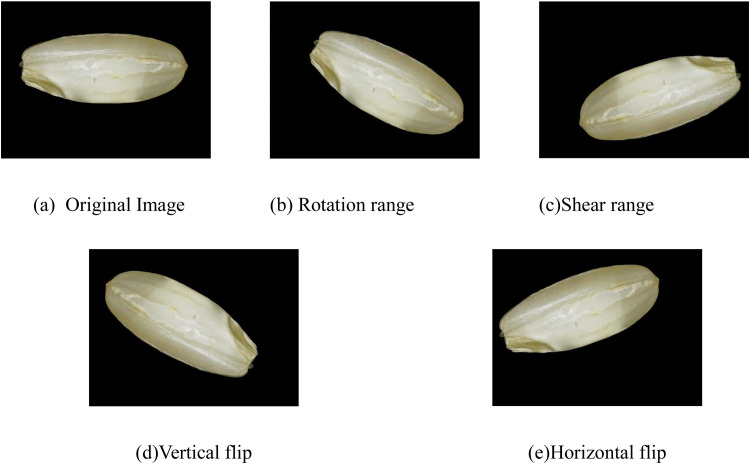
Augmented sample images demonstrating various transformations applied to the original data. (a) Original Image - the baseline image before augmentation, (b) Rotation Range - image rotated within a specified range, (d) Vertical Flip - image flipped along the vertical axis, (e) Horizontal Flip - image flipped along the horizontal axis. These augmentations contribute to model training by enhancing variability in the dataset.

To enhance the variety of the picture dataset for reliable machine learning model training, specific settings in the Image Data Generator were employed. Shear modifications were applied within a predetermined range, and images were subjected to random rotations to vary their orientation. Additionally, images were both horizontally and vertically flipped. The original image dimensions of 640 × 480 were altered through augmentation, resizing them to 624 × 480. For each image, four new images were generated via augmentation, totaling 2,000 images per class. Fig. 5 displays augmentation samples, showcasing various angles, rotations, shears, and flips, thereby enriching the dataset and ensuring a diverse collection of images for enhanced model training.

The parameters used in the augmentation process were inferred using the fill_mode() function of the Keras package in Python. The parameters we used for augmentation were rotation at angles of 45°, 60°, and 90°, width shift range of 0.2, and shear range of 0.2. From 500 original images of each class, we obtained 2000 augmented images. As a result, a total of 76,000 augmented images were included in the dataset. The process of creating and augmenting the dataset prepared it for training machine learning models.

### 4.1 Camera specification

All images were taken with the help of high power 1600× and 1000× digital microscope cameras. The 1600× and 1000× digital microscope cameras boast impressive specifications tailored for precise imaging and analysis in scientific research, education, and industrial applications. With a magnification power of 1600× and 1000× respectively, these cameras offer detailed observations of microscopic subjects. Equipped with high-resolution sensors, they capture images with exceptional clarity and detail. These cameras often feature adjustable LED illumination for optimal lighting control, ensuring accurate imaging in various environments. With user-friendly software interfaces, users can easily capture, analyze, and document images and videos, making them invaluable tools for researchers, educators, and professionals alike in their pursuit of understanding and discovery at the microscopic level. Table 3 shows the detailed camera setup.

**Table 3:** Camera Setup Details.

Color	Black
Size	15*12.5*4.5cm
Sensors	High Performance Photosensitive Chips
Auxiliary Light Source	8 White LED Lamps
Digital zoom	Multistage (1600X and 1000X)
Imaging Distance	Manually adjust 0 infinite distance
Image resolution	Standard 640 × 480
Support systems	WIN XP/VISTA; WN732-bit and 64-bit
Computer Interface	USB20 & USB1.1 Compatibility
Illumination range	0-30000 X-ray control adjustable
Dynamic frame count	30/s Under600 LUX Brightness

## Limitations

The dataset has certain limitations. Although it includes 38 different types of rice, it might not cover every variety of rice that exists, which could limit the model's applicability to other types. Additionally, rice names may vary depending on the region. It is noteworthy that many of the photos in this dataset are of processed or semi-processed rice; therefore, problems involving the classification of raw rice may present difficulties. As for the camera settings and resolutions, the background and lighting of the rice kernels may be a little different.

## Ethics Statement

In this article, there is no research involving human or animal subjects conducted by the authors. The datasets used in this article are publicly accessible. Proper citation of these datasets is important when using them.

## CRediT Author Statement

**Md Tahsin:** Writing, Data curation, Investigation, Augmentation; **Md. Mafiul Hasan Matin**: Data analysis, Supervision, Writing – Review & Editing; **Mashrufa Khandaker:** Data curation; **Redita Sultana Reemu:** Data curation; **Mehrab Islam Arnab:** Data collection; **Mohammad Rifat Ahmmad Rashid:** Supervision, Validation; **Md Mostofa Kamal Rasel:** Supervision, Review & Editing; **Mohammad Manzurul Islam:** Supervision, Validation; **Maheen Islam:** Supervision, Review & Editing; **Md. Sawkat Ali**: Supervision, Validation, Review & Editing.

## Data Availability

Mendeley DataAn Extensive Image Dataset for Classifying Rice Varieties in Bangladesh (Original data).
